# Occurrence and Properties of Thiosilvatins

**DOI:** 10.3390/md17120664

**Published:** 2019-11-26

**Authors:** Maria Michela Salvatore, Rosario Nicoletti, Marina DellaGreca, Anna Andolfi

**Affiliations:** 1 Department of Chemical Sciences, University of Naples ‘Federico II’, 80126 Naples, Italy; mariamichela.salvatore@unina.it (M.M.S.); dellagre@unina.it (M.D.); 2 Council for Agricultural Research and Economics, Research Centre for Olive, Citrus and Tree Fruit, 81100 Caserta, Italy; rosario.nicoletti@crea.gov.it; 3 Department of Agriculture, University of Naples ‘Federico II’, 80055 Portici, Italy

**Keywords:** thiosilvatins, secondary metabolites, diketopiperazines, epipolythiodioxopiperazines, fungi, biological activities

## Abstract

The spread of studies on biodiversity in different environmental contexts is particularly fruitful for natural product discovery, with the finding of novel secondary metabolites and structural models, which are sometimes specific to certain organisms. Within the large class of the epipolythiodioxopiperazines, which are typical of fungi, thiosilvatins represent a homogeneous family that, so far, has been reported in low frequency in both marine and terrestrial contexts. However, recent observations indicate that these compounds have been possibly neglected in the metabolomic characterization of fungi, particularly from marine sources. Aspects concerning occurrence, bioactivities, structural, and biosynthetic properties of thiosilvatins are reviewed in this paper.

## 1. Introduction

A huge chapter on research on biodiversity is represented by studies concerning the biochemical properties of the manifold organisms which are part of natural ecosystems. Novel secondary metabolites are continuously discovered, disclosing a surprising chemodiversity in terms of both structural and biosynthetic aspects. Although most classes of compounds are spread throughout the several kingdoms of nature, a certain specificity results in some cases.

An example in this respect is represented by the epipolythiodioxopiperazines (ETPs), so far only reported from fungi [1]. ETPs are a large and structurally diverse class of bioactive secondary metabolites originating from diketopiperazines and characterized by the presence of a disulfide bridge or a polysulfide dioxopiperazine six-membered ring. Due to their bioactivities, ETPs are receiving attention in recent years [2]. 

**Figure 1:**
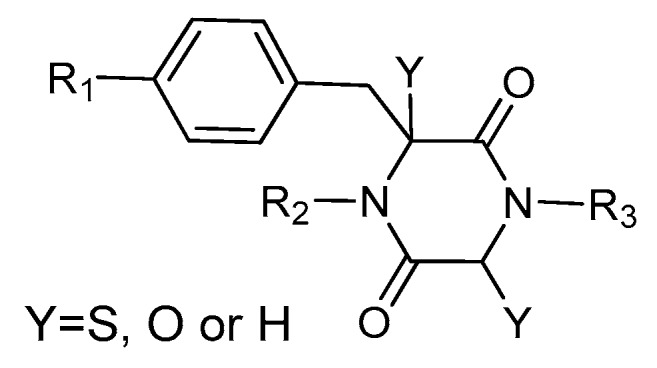
Basic structure of thiosilvatins.

This review is focused on thiosilvatins, a specific family of ETPs resulting from the enzymatic assemblage of two amino acids (i.e., l-tyrosine/l-phenylalanine and glycine), generally integrated with two methylated sulfur atoms. Unlike a related family including hyalodendrin, gliovictin, and their analogues, in this homogeneous group of compounds, the pivotal nitrogen deriving from the aromatic amino acid is not engaged in structural modifications other than methylation (Figure 1).

The finding of natural products displaying this kind of molecular structure only started in the 1980s [3], and even nowadays reports are quite infrequent. The present paper provides a review of the current knowledge concerning occurrence, bioactivities, structural, and biosynthetic aspects of these compounds. To ensure a comparative examination of the several structures reviewed herewith, the name of some compounds was adapted to conform to previously characterized analogs.

## 2. Structures and Chemical Properties

**Figure 2:**
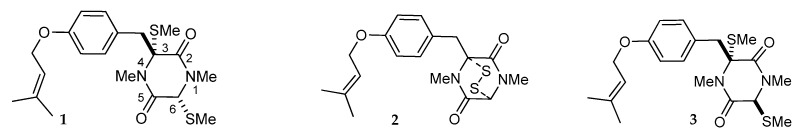
Structures of *cis*-bis(methylthio)silvatin (**1**, NM = 408 U), dithiosilvatin (**2**, NM = 378 U), *trans*-bis(methylthio)silvatin (**3**, NM = 408 U).

(3*R*,6*R*)-1,4-Dimethyl-3-(4-(3-methyl-2-butenyloxy)benzyl)-3,6-bis(methylthio)piperazine-2,5-dione (**1**), the founder product of this compound series, was isolated for the first time in 1981 along with its deprenyl analogue (**16**) [3]. Subsequently, **1** was named *cis*-bis(methylthio)silvatin when the only sulfur bridged thiosilvatin, dithiosilvatin (**2**), was characterized and submitted to a reductive methylation giving **1** and its epimer in C-6 (**3**) [4] (Figure 2).

The most relevant structural modifications observed in this class of compounds are in the number of sulfurs, the degree of methylation of heteroatoms, and the presence of a dimethyl allylic chain (Table 1). A controversial issue concerning thiosilvatins could be represented by nomenclature. In fact, several compounds were inconsistently designated with trivial names, abbreviations, or according to the IUPAC standards, which generally are not linked to the name of the founder compound. For instance, Sch 54794 (**4**) and Sch 54796 (**5**) have the same structures and stereostructures of **1** and **3**, but their amino functions are not methylated; consequently, they could be respectively named *cis*- and *trans*-dinor-bis(methylthio)silvatin [5]. Likewise, *cis*-3-(4-hydroxybenzyl)-1,4-dimethyl-3,6-bis(methylthio)-2,5-piperazinedione (**16**) and *trans*-6-(4-hydroxybenzyl)-1,4-dimethyl-3,6-bis(methylthio)piperazine-2,5-dione (**17**) were named in the original manuscripts according to IUPAC standards [3,6], but they could be easily named respectively *cis*- and *trans*-deprenyl-bis(methylthio)silvatin.

Many difficulties in nomenclature derive from the stereostructural aspects. Concerning compounds **25**–**27**, they were not named in the original manuscript [7]; however, a trivial name can be assigned according to their structurally related compound bilain B. However, the absence of a complete stereostructure determination prevents clarifying if compound **25** is actually its epimer or rather its diasteroisomer.

In recent years, names of some new compounds, such as saroclazines, fusaperazines, and bilains, were assigned considering their sources, rather than referring to their structural relationships. For the reasons explained above, in Table 1 some compounds have also been renamed according to the founder compound.

In general, the biosynthesis of secondary metabolites is stereospecific. In fact, the stereochemistry of chiral carbons in the dioxopiperazinic ring of thiosilvatins is essentially 3*R*,6*R*, even if these compounds display some structural differences. This is observed in strains belonging to unrelated species, such as *Fusarium chlamydosporum* [8], *Penicillium waksmanii* [9], *Penicillium brevicompactum* [10], *Trichoderma virens* [3,11], all producing thiosilvatins with the same stereostructure [i.e., *cis*-bis(methylthio)silvatin (**1**) and its deprenyl analogue (**16**)] (Table 1, Table 2 and Table 3).

**Figure 3:**
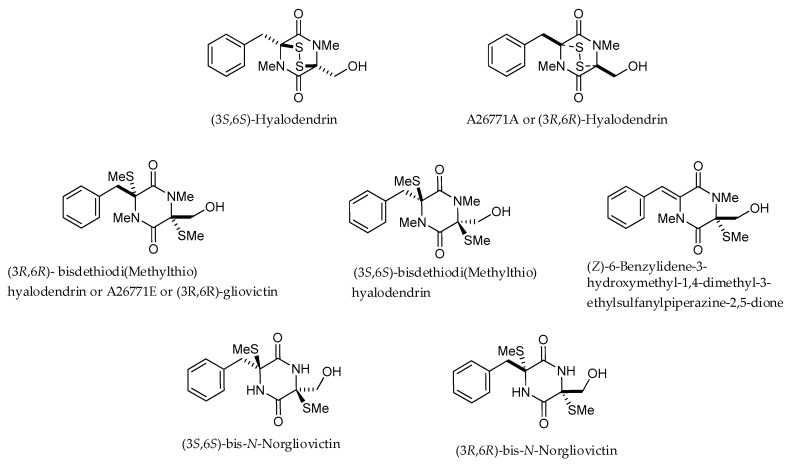
Hyalodendrins and gliovictins.

On the other hand, this is not a common trend in compounds belonging to the ETPs class [2]. In fact, both stereoisomers (i.e., 3*R*,6*R* and 3*S*,6*R*) were reported for compounds in the hyalodendrin/gliovictin family, deriving from the amino acids l-phenylalanine and l-serine [12,13,14] (Figure 3). Interestingly, this family also includes a compound named vertihemiptellide A, representing the first dimer resulting from the formation of disulfide bridges between two hyalodendrin units [15].

## 3. Fungal Sources

As introduced above, so far ETPs have been only reported from fungi. More specifically, thiosilvatins have been detected as secondary metabolites of 22 strains belonging to 17 taxa that occupy different geographic and climatic zones, terrestrial and marine habitats, and are associated with different substrates/hosts (Table 2 and Table 3). With the exception of a single taxon in the Basidiomycota, that is *Coriolus (=Irpex*) *consors*, all the other strains are representative of taxa in the Ascomycota. Particularly, they belong to the Sordariomycetes (order Hypocreales, 7 strains/6 taxa; order Xylariales, 1 strain/taxon), and to the Eurotiomycetes (14 strains/9 taxa, all of them in the Eurotiales). Twelve strains, that is more than half of the total number, belong to the genus *Penicillium*, well known for its widespread occurrence in every ecological context including the sea [16]. The species *Penicillium crustosum* and *T. virens* include strains from both kind of sources.

**Figure 4:**
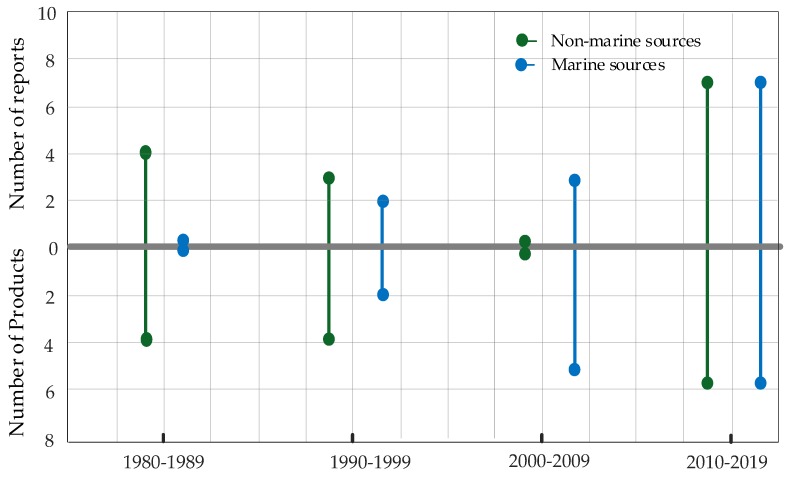
Head to tail comparison of number of reports dealing with thiosilvatins and number of new products obtained from marine and non-marine sources.

With reference to the specific compounds, **1** undoubtedly represents the most common product of this family, having been reported as a secondary metabolite of about 2/3 of the strains, while its *trans* stereoisomer has been detected in just four of these strains, both marine and terrestrial. Compounds **4**, **5**, **12**, **14** and **16** were also obtained from strains from both environments. Among the rest, compounds **6**, **7**, **11**, **15** and **17**–**24** have been reported from just a single strain of marine origin, while compounds **2**, **8**–**10**, **13,** and **25**–**27** have been only found in terrestrial strains. These data could be indicative of a relatively higher chemodiversity characterizing marine strains, also considering that reports from marine sources only started in 1998 when there were already four strains and eight products known from terrestrial sources (Figure 4). Since 1998, the new products discovered from marine fungi more than doubled those obtained from non-marine strains. Moreover, in the last two years there were five reports concerning new thiosilvatins-producing strains from marine sources compared to two from terrestrial sources, which might imply that a more widespread occurrence at sea is likely to be disclosed as investigations concerning marine fungal strains progress. Finally, no comparison can be made between strains of the same species (*P. crustosum* and *T. virens*) obtained from both marine and terrestrial sources, whose secondary metabolite profiles do not match, or share single compounds. This could be interpreted not only in terms of intraspecific variation, but also as a consequence of the different culturing and extraction procedures. Moreover, it must also be considered that detection of some compounds is often impaired by their presence in low quantities, or by inherent difficulties in the identification depending on their infrequent occurrence. However, the finding of two species from both marine and terrestrial sources within such a limited strain sample supports a recently-consolidated inference that most fungal species are able to thrive in different environmental conditions, obliterating the old misconception that the occurrence of specialized taxa occurs in either marine or non-marine contexts [16,31,32].

## 4. Proposed Biosynthetic Pathways for Thiosilvatins

The biosynthesis of ETPs involves non-ribosomal peptide synthetases (NRPSs), multi-domain enzymes controlling all activities required to incorporate constituents into their products, and a range of associated enzymes [33,34]. In fact, the non-ribosomal pathway is frequently used by microorganisms to produce a wide range of structurally diverse secondary metabolites [35].

In general, the genes that encode enzymes for secondary metabolite biosynthesis are clustered in the fungal genome [36]. Some ETP gene clusters, such as the ones involved in sirodesmin and gliotoxin biosynthesis, were identified by generating mutations in these genes and analyzing secondary metabolite profiles of the resultant mutants. In fact, the gene deletions may result in abrogation of the biosynthetic pathway. The comparative analysis of many fungal genome sequences has displayed similarities between the gliotoxin and sirodesmin clusters, proving the conservation of the main biosynthetic genes in the ETP clusters. It is thus likely that similar core enzymes are responsible for the biosynthesis of the ETP backbone, but the structural diversity depends on other genes that appear only in some clusters, many of them remaining to be fully identified [37,38,39,40,41]. 

**Figure 5:**
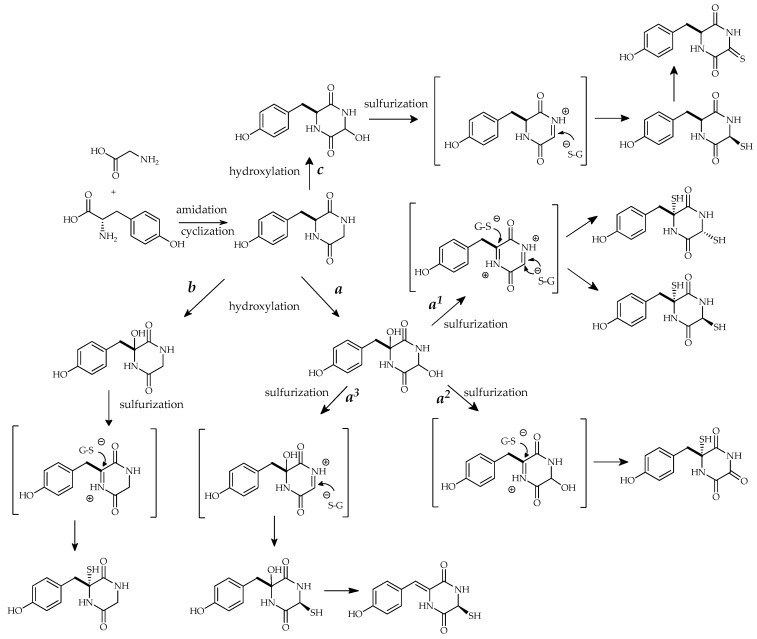
Proposed biosynthetic pathways for thiosilvatins. S-G represents glutathione.

Based on these pieces of evidence, the thiosilvatin biosynthesis was predicted according to the one reported for gliotoxin [1,42,43]. In fact, similar to other ETPs, thiosilvatins derive from the condensation of two amino acids which can be further altered by epimerization, methylation, or cyclization. The origin and mechanism of incorporation of the sulfur atoms into the dipeptide are unclear, and according to different hypotheses they could be derived from methionine, cysteine, sodium sulfate, or glutathione. Particularly, the formation of a diiminium intermediate followed by nucleophilic attack of the cysteine thiolate residue of glutathione is possibly involved (Figure 5) [2,39]. In order to justify the presence of C-6 epimers on the piperazine ring, two possible mechanisms of nucleophilic attack have been proposed (Figure 5, reaction mechanism a^1^).

An alternative biosynthetic pathway, even if less credited, has been proposed for the sulfurization of diketopiperazines. Due to the slow rate of dipeptide cyclization, the sulfur insertions and further chemical transformations might occur while the linear dipeptide is still covalently bound to the NRPS [44].

A different biosynthetic pathway could be possible for monosulfurate compounds, which represent an extensive group in the thiosilvatin compounds series, as exemplified in the reaction mechanisms a^2^, a^3^, and b in Figure 5. Sulfur insertion could happen on C-3 or C-6 of the hydroxypiperazine ring in the iminium intermediate followed by hydroxyl oxidation or water elimination to obtain precursors of **12**, **13** and **14**, **15**.

Silvathione (**8**) might have a different biosynthetic pathway with monoimmyl intermediate which involves C-6 and ^1^N (Figure 5, reaction mechanism c).

Further chemical transformations (e.g., methylation, oxidation) possibly occur on the backbone of thiosilvatins in order to obtain an ample variety of natural products. In fact, nitrogen and oxygen atoms can be methylated, while the phenolic hydroxyls are frequently prenylated (i.e., **1**–**15**, **21**–**24**).

## 5. Biological Activities

Although no conclusive demonstration has been obtained yet, the opinion is prevalent that ETPs are important for the producing strains in the interaction with other organisms. These compounds have been reported for a wide array of bioactive properties, including antibiotic, antiviral, cytotoxic and anti-inflammatory effects. Bioactivities basically depend on thiol-disulphide exchange reactions, and the relative effects of the single compounds are considered to be somehow related to the oxidation/reduction status of the sulfurs [1]. 

Unlike the homologue hyalodendrin/gliovictin family, a few members of which have been more extensively investigated with reference to their antibiotic and antiproliferative properties, and mechanisms of action [15,45,46], for thiosilvatins the available data are still quite preliminary for drawing a clear judgment concerning their biological activity and opportunities for pharmaceutical exploitation.

No antifungal properties could be evidenced in assays carried out with *cis-*bis(methylthio)silvatin on *Parastagonospora* (*Septoria*) *nodorum* [19], and yeast strains of *Candida albicans* [30] and *Saccharomyces cerevisiae* [29]. Also, this compound and bilain A did not display antihelmintic activity against the barber’s pole worm (*Haemonchus contortus*), a common parasitic nematode of ruminants [19], while *cis*-deprenyl-bis(methylthio)silvatin (**16**) and *trans*-deprenyl-bis(methylthio)silvatin (**17**) did not show toxic effects on *Artemia salina* and four marine phytoplankton species (*Chattonella marina, Heterosigma akashiwo, Karlodinium veneficum,* and *Prorocentrum donghaiense*) at a concentration of 100 μg mL^−1^ [6].

Assays concerning antibacterial activity mostly provided negative results, too. In fact, **16** and **17** were inactive against five marine-derived pathogenic Gram-negative bacteria (*Vibrio parahaemolyticus, V. anguillarum, V. harveyi, V. splendidus*, and *Pseudoalteromonas citrea*) in an agar disk-diffusion assay at a dose of 20 μg/disk [6]. No effects were observed for **1** against *Escherichia coli* and *Bacillus subtilis* [19] and, together with its *trans* stereoisomer (**3**), fusaperazine E (**14**) and *trans*-dinor-bis(methylthio)silvatin (**5**), against *Enterococcus faecalis* [27]. However, more recently, some extent of antibacterial properties by *cis-*bis(methylthio)silvatin have been reported against *Staphylococcus aureus* (MIC 43.4 µg mL^−1^) [30], *E. coli* and *B. subtilis* (IC_50_ 30.0 μg mL^−1^) [29].

In line with the recent trend to screen natural products in the aim of finding new anticancer compounds, more circumstantial data are available with reference to the antiproliferative activity against tumor cell lines. In this respect, fusaperazine A (**18**) and **1** exhibited weak cytotoxic activities against P388 murine lymphocytic leukaemia cells (ED_50_ 22.8 and 7.7 µg mL^−1^, respectively) [8], confirming previous findings concerning the latter compound [9]. In another study *cis*-bis(methylthio)silvatin was cytotoxic (0.15 µM) on NS-1 mouse myeloma cells, while bilain A (**22**) was inactive [19]. Again on P388 cells, *cis*-dinor-bis(methylthio)silvatin (**4**) exhibited weak cytotoxic activity (ED_50_ 21.5 µg mL^−1^), whereas its analogue **5** was inactive along with fusaperazine B (**11**) and **16** [8]. Afterwards, **4** and **5** were found to remarkably inhibit the growth of two human cell lines HEp2 (larynx carcinoma) and HepG2 (liver carcinoma) [22]. Citriperazines A and B (**19**, **20**) did not exhibit cytotoxic activity against three human prostate cancer cells (22Rv1, PC-3 and LNCaP) at concentrations up to 100 µM, also without any significant effect on cell cycle progression [22]. Cytotoxic effects have been also reported for saroclazine B (**7**) against HeLa (cervyx uteri carcinoma) cells (IC_50_ 4.2 µM) [24], and fusaperazine F (**15**) against the K562 (chronic myelogenous leukemia) cell line (IC_50_ 12.7 μM) [21].

In a quite peculiar assay carried out on zebrafish larvae, **1** and 6-oxo-methylthiosilvatin (**12**) promoted gastrointestinal motility via acting on the cholinergic nervous system, while bilains D–F (**25**–**27**) lacking the double bond in the lateral chain were inactive [7].

Finally, on account of the platelet-activating factor (PAF) inhibitory effects also known for other diketopiperazines [47], a weak activity was displayed by compound **5** in the PAF assay (IC_50_ 50 µM), while the related **4** was inactive [5].

## 6. Conclusions

As introduced above, literature concerning the occurrence and properties of thiosilvatins is not extensive. Although half of the reports refer to strains of *Penicillium*, the available data show that biosynthetic aptitude for these compounds can be found in distantly related fungal species, in line with what is known for the homologue hyalodendrin/gliovictin family, other ETPs, and many mycotoxins. Actually, this biological phenomenon, known under the name of synapomorphy, is quite difficult to explain in phylogenetic terms, since it would imply that the genetic base encoding for biosynthesis of these secondary metabolites was acquired or lost many times along with the separation of lineages during the evolution of fungi. However, as the work on genome sequencing of fungi progresses, the evidence is accumulating that biosynthesis of many classes of mycotoxins is controlled by clustered genes. And the discovery that fungi may exchange gene clusters through the so-called horizontal gene transfer (HGT) has disclosed a more reasonable biological explanation, according to which fungal species thriving in the same ecological niche or sharing the same substrate may somehow establish a successful interaction at the genetic level resulting in modification of their metabolome [48,49].

In this respect, the occurrence in clusters of genes involved in the biosynthesis of ETPs has been demonstrated in the case of some major members of this class, such as gliotoxin [50], sirodesmin [37], and verticillin [51]. Moreover, gene clusters with all eight genes encoding for the common ETP moiety have been found in several unrelated ascomycetes species [1]. Following assumptions in comparative genomics, more recent evidence indicates that such a cluster may be present even in fungal species which so far have not been reported for production of these compounds [52].

The accumulation of data concerning metabolomics of fungal strains/species is fundamental in order to provide more circumstantial support to this theory and to shed light on the circumstances which make HGT possible. In this regard, thiosilvatins appear to represent a meaningful group of compounds, characterized by a uniform molecular model, possibly reflecting a definite biosynthetic scheme.

More prompts for developing investigations on the biological properties of thiosilvatins derive from the availability of more refined bioassays able to elucidate the effects of compounds displaying low levels of cytotoxicity. An interesting example in this respect is provided by the finding of a diketopiperazine derivative inhibiting prion replication in the micromolar range, which introduces these compounds as a promising lead scaffold in the search of products against these problematic disease determinants [53]. Finally, the very recent finding from a strain of *Penicillium roqueforti* from blue cheese [29] introduces the opportunity to better investigate the effects deriving from a dietary intake of *cis*-bis(methylthio)silvatin, also in association with the roquefortines and other bioactive products reported from this species of biotechnological relevance [54,55].

## Figures and Tables

**Table 1:** Thiosilvatins reported as natural products. Compound names proposed in this review are underlined.

Code	Compound	Structure	Formula, Nominal Mass (U)
	***N*-Demethyl analogues**		
**4**	Sch 54794; *cis*-dinor-bis(methylthio)silvatin	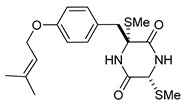	C_18_H_24_N_2_O_3_S_2_ 380
**5**	Sch 54796; *trans*-dinor-bis(methylthio)silvatin	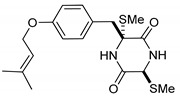	C_18_H_24_N_2_O_3_S_2_ 380
**6**	Saroclazine A	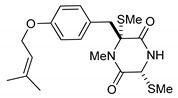	C_19_H_26_N_2_O_3_S_2_ 394
**7**	Saroclazine B	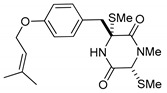	C_19_H_26_N_2_O_3_S_2_ 394
	**Dethio analogues**		
**8**	Silvathione	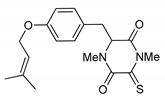	C_18_H_22_N_2_O_3_S 346
**9**	3-(4-(3-Methyl-2-butenyloxy)benzyl)-3-(methylthio)-2,5-piperazinedione; dinor-methylthiosilvatin	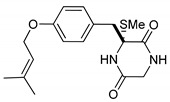	C_17_H_22_N_2_O_3_S 334
**10**	6-Hydroxy-3-(4-(3-methyl-2-butenyloxy)benzyl)-3-(methylthio)piperazine-2,5-dione; dinor-hydroxy-methylthiosilvatin	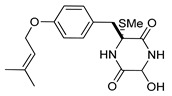	C_17_H_22_N_2_O_4_S 350
**11**	Fusaperazine B (relative stereochemistry)	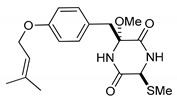	C_18_H_24_N_2_O_4_S 364
**12**	1,4-Dimethyl-6-(4-(3-methyl-2-butenyloxy)benzyl)-6-methylsulfanyl-piperazine-2,3,5-trione; 6-oxo-methylthiosilvatin	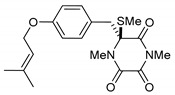	C_19_H_24_N_2_O_4_S 376
**13**	6-(4-(3-Methyl-2-butenyloxy)benzyl)-6-methylsulfanyl-piperazine-2,3,5-trione; dinor-6-oxo-methylthiosilvatin	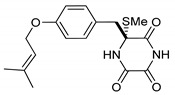	C_17_H_20_N_2_O_4_S 348
**14**	Fusaperazine E	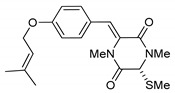	C_19_H_24_N_2_O_3_S 360
**15**	Fusaperazine F	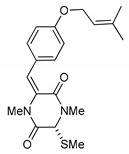	C_19_H_24_N_2_O_3_S 360
	**Deprenyl analogues**		
**16**	*cis*-3-(4-Hydroxybenzyl)-1,4-dimethyl-3,6-bis(methylthio)-2,5-piperazinedione; *cis*-deprenyl-bis(methylthio)silvatin	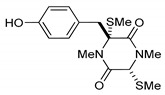	C_15_H_20_N_2_O_3_S_2_ 340
**17**	*trans*-6-(4-Hydroxybenzyl)-1,4-dimethyl-3,6-bis(methylthio)piperazine-2,5-dione; *trans*-deprenyl-bis(methylthio)silvatin	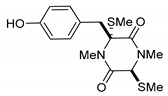	C_15_H_20_N_2_O_3_S_2_ 340
**18**	Fusaperazine A	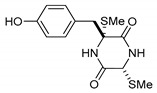	C_13_H_16_N_2_O_3_S_2_ 312
**19**	Citriperazine A	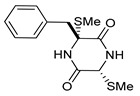	C_13_H_16_N_2_O_2_S_2_ 296
**20**	Citriperazine B	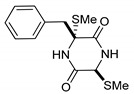	C_13_H_16_N_2_O_2_S_2_ 296
	**Prenyl chain modified analogues**		
**21**	*cis*-3-(4-(4-Hydroxy-3-methyl-2-butenyl)oxy)benzyl)-1,4-dimethyl-3,6-bis(methylthio)piperazine-2,5-dione; bis-(methylthio)silvatinol	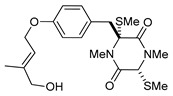	C_20_H_28_N_2_O_4_S_2_ 424
**22**	Bilain A	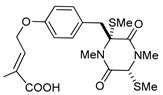	C_20_H_26_N_2_O_5_S_2_ 438
**23**	Bilain B	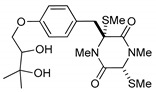	C_20_H_30_N_2_O_5_S_2_ 442
**24**	Bilain C	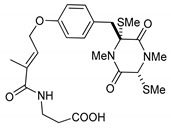	C_23_H_31_N_3_O_6_S_2_ 509
**25**	Bilain D	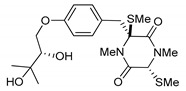	C_20_H_30_N_2_O_5_S_2_ 442
**26**	Bilain E	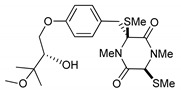	C_21_H_32_N_2_O_5_S_2_ 456
**27**	Bilain F	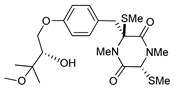	C_21_H_32_N_2_O_5_S_2_ 456

**Table 2:** Marine-derived fungal strains producing thiosilvatins.

Species (Strain)	Source	Geographic Origin	Compound Code	Ref.
*Cordyceps javanicus*^1^ (961331)	*Jaspis* cf. *coriacea* (sponge)	Fiji	**1**, **3**	[17]
*Fusarium chlamydosporum* (OUPS-N124)	*Carpopeltis affinis* (red alga)	Japan	**1**, **4**, **5**, **11**, **16**, **18**	[8]
*Nigrospora* sp. (PSU-F12)	*Annella* sp. (gorgonian)	Similan Islands (Thailand)	**5**	[18]
*Penicillium bilaiae* (MST-MF667)	Boat ramp	Huon estuary, Tasmania (Australia)	**1**, **22**, **23**, **24**	[19]
*Penicillium commune* (518)	*Muricella abnormalis* (gorgonian)	Danzhou, Hainan (China)	**1**	[20]
*Penicillium crustosum* (HDN153086)	Sediment	Prydz Bay (Antarctica)	**1**, **3**, **15**	[21]
*Penicillium* sp. (KMM 4672)	*Padina* sp. (brown alga)	Vietnam	**19**, **20**	[22]
*Penicillium* sp. (2556)	Mangrove plant	China	**4**, **5**	[23]
*Penicillium waksmanii* (OUPS-N133)	*Sargassum ringgoldianum* (brown alga)	Japan	**1**, **16**, **21**	[9]
*Sarocladium kiliense* (HDN11-84)	Rhizosphere soil of *Thespesia populnea* (mangrove)	Guangxi (China)	**1**, **6**, **7**, **12**	[24]
*Trichoderma virens* (Y13-3)	*Gracilaria vermiculophylla* (red alga)	Yangma Island (China)	**16**, **17**	[6]

^1^ This strain identified with the older species name of *Paecilomyces* cf. *javanica* in the original report.

**Table 3:** Fungal strains from non-marine sources producing of thiosilvatins.

Species (Strain)	Source	Geographic Origin	Compound Code	Ref.
*Aspergillus silvaticus* (IFO8173)	Soil	Tafo (Ghana)	**8**, **2**	[4]
*Coriolus (=Irpex) consors* (ATCC11574)	ATCC collection		**1**, **3**	[25]
*Penicillium amphipolaria* (DAOM695760)	Soil	Quartermain Mountains (Antarctica)	**14**	[26]
*Penicillium brevicompactum*	Contaminant in culture of *Ceratocystis ulmi* (plant pathogenic fungus)	Edmonton (Canada)	**1**, **9**, **10**, **16**	[10]
*Penicillium crustosum* (VR4)	*Viguiera robusta* (plant)	Brazil	**1**, **3**, **14**	[27]
*Penicillium crustosum* (MK285663)	Fruiting body of *Isaria cicadae* (entomopathogenic fungus)	Sichuan province (China)	**1**, **13**, **25**, **26**, **27**	[7]
*Penicillium crustosum* (YN-HT-15)	Red soil	Yunnan (China)	**12**, **13**	[28]
*Penicillium roqueforti* (ATCC10110)	Blue cheese	USA	**1**	[29]
*Penicillium* sp.	Endophytic in *Pinellia ternata* (plant)	Nanjing (China)	**1**	[30]
*Tolypocladium* sp.	*Quercus virginiana* (plant)	Tamalupas (Mexico)	**1**, **4**, **5**, **9**	[5]
*Trichoderma virens*^1^ (CMI101525)	Soil	California, USA	**1**, **16**	[3,11]

^1^ This strain identified with the older species name of *Gliocladium deliquescens* in the original report.
